# CHK2 Promotes Metabolic Stress-Induced Autophagy through ULK1 Phosphorylation

**DOI:** 10.3390/antiox11061166

**Published:** 2022-06-14

**Authors:** Ran Guo, Shan-Shan Wang, Xiao-You Jiang, Ye Zhang, Yang Guo, Hong-Yan Cui, Qi-Qiang Guo, Liu Cao, Xiao-Chen Xie

**Affiliations:** 1 Department of Orthopedics, Shengjing Hospital of China Medical University, Shenyang 110004, China; sj_guoran@163.com; 2 College of Basic Medical Science, Health Sciences Institute, Key Laboratory of Medical Cell Biology, Ministry of Education, China Medical University, Shenyang 110122, China; shanshanwang0128@gmail.com (S.-S.W.); 18204509380@163.com (X.-Y.J.); 18002471466@163.com (Y.Z.); cmuguoyang@163.com (Y.G.); c2447076105@163.com (H.-Y.C.); 3 Liaoning Provincial Key Laboratory of Endocrine Diseases, Department of Endocrinology and Metabolism, Institute of Endocrinology, The First Affiliated Hospital of China Medical University, Shenyang 110001, China

**Keywords:** autophagy, ULK1, CHK2, oxidative stress, ROS

## Abstract

Reactive oxygen species (ROS) act as a signaling intermediate to promote cellular adaptation to maintain homeostasis by regulating autophagy during pathophysiological stress. However, the mechanism by which ROS promotes autophagy is still largely unknown. Here, we show that the ATM/CHK2/ULK1 axis initiates autophagy to maintain cellular homeostasis by sensing ROS signaling under metabolic stress. We report that ULK1 is a physiological substrate of CHK2, and that the binding of CHK2 to ULK1 depends on the ROS signal and the phosphorylation of threonine 68 of CHK2 under metabolic stress. Further, CHK2 phosphorylates ULK1 on serine 556, and this phosphorylation is dependent on the ATM/CHK2 signaling pathway. CHK2-mediated phosphorylation of ULK1 promotes autophagic flux and inhibits apoptosis induced by metabolic stress. Taken together, these results demonstrate that the ATM/CHK2/ULK1 axis initiates an autophagic adaptive response by sensing ROS, and it protects cells from metabolic stress-induced cellular damage.

## 1. Introduction

Oxidative damage to cellular biomolecules caused by excess reactive oxygen species (ROS) is the root cause of apoptosis and a potential factor leading to a range of pathologies, including neurodegenerative diseases, atherosclerosis, and aging processes [1,2]. In recent years, an increasing number of studies have shown that ROS appear to be induced in response to pathophysiological stress and act as signaling intermediates that promote adaptive cellular responses to maintain homeostasis [3,4]. Autophagy, a multistep lysosomal degradation pathway that supports nutrient recycling and metabolic adaptation, has been implicated as an essential biological behavior for maintaining homeostasis [5]. ROS have been widely recognized as being central signaling molecules that induce autophagy under various stimuli [4,6]. However, the mechanism by which ROS promote autophagy is still largely unknown.

Cell cycle checkpoint kinase 2 (CHK2) is an evolutionarily highly conserved serine/threonine-protein kinase that was initially identified as a vital transducer in the DNA damage response (DDR) [7]. When DNA damage occurs, ataxia-telangiectasia mutated (ATM) phosphorylates CHK2 at threonine 68. After the phosphorylation of CHK2 inactive monomers at T68 by ATM, dimers form and undergo subsequent autophosphorylation, and may also serve as substrates for other protein kinases. Therefore, the phosphorylation of CHK2 T68 is considered to be a prerequisite for CHK2 to be activated and to perform signaling functions, such as cell cycle arrest, DNA repair, cell survival, proliferation, and cell death [8,9]. In recent years, the ATM-CHK2 signaling pathway, which can act as a sensor of ROS and participate in autophagy to maintain cell homeostasis, has received more attention [10,11]. The oxidation of ATM (Cys2991) directly induces ATM activation (phospho-ATM Ser1981) in the absence of DNA DSBs and the MRN complex [12]. In response to elevated ROS, ATM activates TSC2 through the LKB1/AMPK signaling pathway to inhibit mTORC1, and it promotes autophagy [13]. Our previous study found that elevated levels of ROS induced by glucose deprivation and hypoxia can promote autophagy to maintain cellular homeostasis by activating the ATM/CHK2/Beclin 1 axis [10]. However, DDR signaling as a direct crosstalk mechanism between ROS and autophagy remains to be further explored.

The serine/threonine-protein kinase ULK1 is the homologous protein of yeast Atg1 in mammalian cells [14]. It forms a complex with Atg13, FIP200, and Atg101, and plays a vital role in autophagy [15,16,17]. Phosphorylation of ULK1 is critical in autophagy regulation [18]. Under adequate nutrient conditions, mTOR phosphorylates ULK1 and Atg13, resulting in the inactivation of the ULK1 complex. Under starvation conditions, by inhibiting the activity of mTORC1, the inhibitory effect on ULK1 is relieved, and the kinase activity of ULK1 is enhanced, which can further phosphorylate Atg13 and FIP200 to initiate the occurrence of autophagy [19]. In addition, AMPK can promote autophagy by phosphorylating multiple serine/threonine sites in the intermediate domain of ULK1 (including serines 317, 467, 555, 637, and 777, and threonine 574) [20,21,22,23,24]. However, the regulatory mechanism for how ROS signaling promotes autophagy through ULK1 remains unclear.

Here, we found that the autophagy-related protein ULK1 is a new interaction protein of CHK2, and that its binding depends on the accumulation of ROS caused by metabolic stress. CHK2 phosphorylates ULK1 at serine 556 to promote autophagy and inhibit cell apoptosis under metabolic stress. Our findings establish that the ATM/CHK2/ULK1 signaling pathway initiates an autophagic adaptive response by sensing ROS, and it protects cells from metabolic stress-induced cellular damage.

## 2. Materials and Methods

### 2.1. Plasmids Constructs, Cell Culture, and Viral Infection

ULK1 expression plasmids were kindly provided by Xin Pan (National Center of Biomedical Analysis, Beijing, China). ULK1 was subcloned into pGEX-5X-1. CHK2 (Addgene, no. 41901, Watertown, MA, USA) was subcloned into pcDNA3.1 with an amino-terminal 3× Flag tag. ULK1^Mut^ constructs, ULK1^S556A^ and ULK1^S556D^, and CHK2^Mut^ constructs, CHK2^T68A^ and CHK2^T68D^ were created using site-directed mutagenesis.

HEK293T and HEK293 cells were cultured in Dulbecco’s modified Eagle’s medium (DMEM) containing 10% fetal bovine serum (FBS) and supplemented with 100 μg/mL penicillin/streptomycin. H1299 and HCT116 cells were cultured in RPMI1640 medium containing 10% FBS. The cells were cultured in standard conditions (37 °C, 5% CO_2_). N-acetyl cysteine (NAC) was purchased from Sigma (Beijing, China). CHK2 Inhibitor II (2-(4-(4-Chlorophenoxy)phenyl)-1H-benzimidazole-5-carboxamide hydrate) was purchased from Sigma (C3742). CHK2 inhibitor II specifically inhibits CHK2 phosphorylation at Thr68 and is specific for the inhibition of CHK2 activity. The ATM inhibitor KU-55933 (2-(4-Morpholinyl)-6-(1-thianthrenyl)-4H-Pyran-4-one) was from Sigma (SML1109).

ShRNA against CHK2, and ULK1 lenti-virus were purchased from GeneChem (Shanghai, China). The CHK2 sequence was 5′- ACAGATAAATACCGAACAT -3′ and the ULK1 sequence was 5′- CACGCCATCTCCTCAAGTT -3′. GFP-mCherry-LC3 lenti-virus was purchased from Syngentech (Beijing, China).

### 2.2. Western Blotting

For the Western blot analysis, cells were collected and lysed with lysis buffer (50 mM Tris, pH 7.4, 150 mM NaCl, 1 mM EDTA, 1% NP40, 1% Triton X-100, and 0.25% sodium deoxycholate) containing protease inhibitor cocktail, on ice for 30 min. Afterwards, the cells were vortexed every 10minutes and the lysates were centrifuged at 13,000× *g* for 20 min at 4 °C. Then, the quantified proteins were subjected to SDS-PAGE electrophoresis, after which the proteins were transferred to nitrocellulose, and the membranes were immunoblotted with the indicated primary antibodies. Antibody information and usage are as follows: rabbit anti-ULK1 (D9D7) mAb #6439 (1:1000, CST), rabbit anti-phospho-ULK1 (D1H4) mAb#5869 (S555 (mouse), S556 (human)) (1:1000, CST), rabbit anti-ATM (D2E2) mAb #2873 (1:1000, CST), rabbit anti-phospho-ATM (Ser1981) (D25E5) mAb #13050 (1:1000, CST), rabbit anti-CHK2 mAb #2662 (1:1000, CST), rabbit anti-phospho-CHK2 (Thr68) mAb (1:1000, CST), rabbit anti-p62/SQSTM1 P0067(1:2000, Sigma), rabbit anti-LC3A/B #4108 (1:1000, CST), mouse anti-Flag SG4110-16 (1:1000, Shanghai Genomics Technology, Shanghai, China), and mouse anti-tubulin AC012 (1:2000, ABclonal Technology, Woburn, MA, USA).

### 2.3. Co-Immunoprecipitation

HCT116 cells were transfected with the indicated plasmids using Lipofectamine 3000 (ThermoFisher, L3000008, Waltham, MA, USA). After 24 h of expression, the cells were treated with Earle’s balanced salt solution (EBSS) (GIBCO, Shanghai, China), a nutrient-deprivation medium for 1 h and then lysed with IP lysis buffer. Immune complexes conjugated with primary antibodies to protein A/G beads (Santa Cruz, Dallas, TX, USA) were added to the quantified cell lysates, incubated overnight at 4 °C, and washed three times. Whole cell lysates and precipitation samples were analyzed by Western blot.

### 2.4. In Vitro GST Pull-Down

The protein of GST-ULK1 was induced by IPTG in *E. coli BL21*, and purified by glutathione sepharose4B (GE Healthcare, Chicago, IL, USA) according to the manufacturer’s protocol. At the same time, FLAG-tagged CHK2 was synthesized by using a transcription and translation in vitrokit (Promega, P2221, Madison, WI, USA). The purified protein GST-ULK1 was incubated with FLAG-tagged CHK2 synthesized in vitro, and its direct binding in vitro was detected by Western blot.

### 2.5. In Vitro CHK2 Kinase Assay

Flag-tagged wild-type ULK1 or mutant ULK1 (S556A) was washed three times with kinase buffer (50 mM HEPES, pH 7.4, 10 mM MgCl2, 10 mM MnCl2, and DTT 0.2 mM), and then incubated with CHK2 recombinant human protein (PV3367, ThermoFisher SCIENTIFIC) in kinase reaction buffer (kinase buffer containing 100 μM ATP (Sigma)) at 30 °C for 45 min. Thephosphorylated proteins were subjected to SDS-PAGE electrophoresis, and afterwards, the proteins were transferred to nitrocellulose and the membranes were immunoblotted with the phospho-ULK1 (Ser556) antibody.

### 2.6. Fluorescence Microscopy

HEK293 cells stably expressing GFP-mCherry-LC3 were grown on coverslips, induced by EBSS starvation for 3 h, and then fixed with 4% paraformaldehyde for 20 min. Confocal images were obtained using a 60× oil lens objective on an inverted fluorescence microscope (Nikon, A1RHD25, Japan, Tokyo). The fluorescence assay was performed as described previously [10].

### 2.7. Flow Cytometric Analysis

Flow cytometry analysis of apoptosis was performed according to the manufacturer’s instructions (KeyGENBioTECH, KGA1026, Nanjing, China). In brief, cells were treated with EBSS for 8 h or H_2_O_2_ for 8 h, and then harvested by trypsinization without EDTA. After two washes with PBS, the cells were stained by Annexin V-APC and 7AAD for 30 min and then resuspended in binding buffer solution for FACS analysis.

### 2.8. Statistical Analysis

Statistical comparisons between only two groups were carried out using two-sided *t*-tests. A one-way analysis of variance (ANOVA) was used for multiple-group comparisons. Data are presented as mean ± SEM. We tested data for normality and variance, and considered a *p* value of less than 0.05 as significant. Statistical calculations were performed using GraphPad Prism 5.0.

## 3. Results

### 3.1. ULK1 Is a Physiological Substrate of CHK2

**Figure 1:**
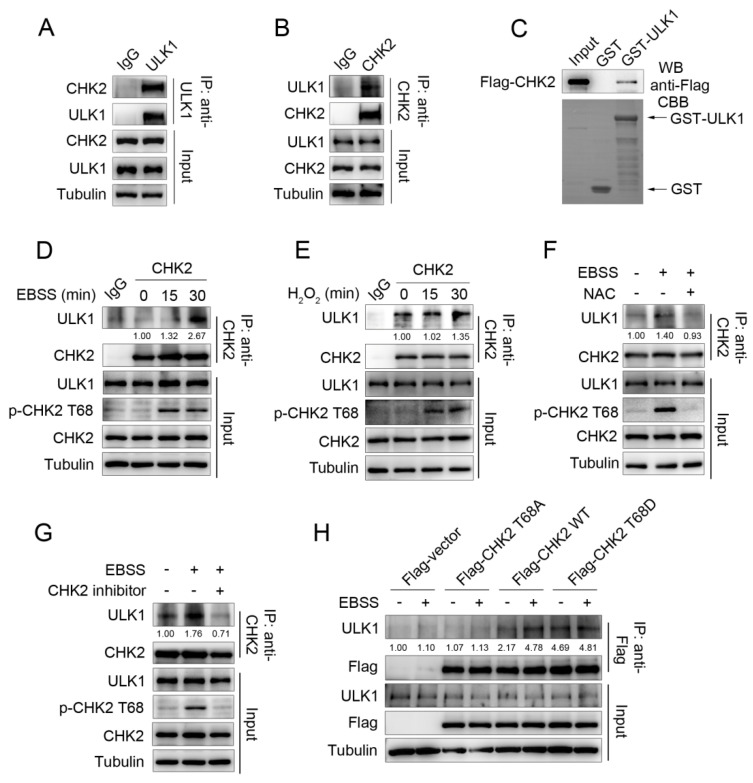
ULK1 is a physiological substrate of CHK2. (**A**,**B**) Immunoprecipitation assays testing the endogenous interaction between CHK2 and ULK1 in HCT116 cells. Lysates were immunoprecipitated with ULK1 (**A**) or CHK2 (**B**) antibody. The immunoprecipitates and lysates were analyzed by Western blot. (**C**) CHK2 binds with ULK1 in vitro. GST pull-down assays were performed by incubating purified GST or GST-ULK1 with invitro-translated flag-tagged CHK2. Arrows indicate GST and GST-ULK1 bands. (**D**) Lysates from HCT116 cells treated with EBSS were immunoprecipitated with CHK2 antibody or rabbit IgG. The immunoprecipitates and lysates were analyzed by Western blot. (**E**) Lysates from HCT116 cells treated with H_2_O_2_ (500 μM) were immunoprecipitated with CHK2 antibody or rabbit IgG. The immunoprecipitates and lysates were analyzed by Western blot. (**F**) Lysates from HCT116 cells pretreated with NAC (SIGMA, A7250, 2 mM) for 3 h and then cultured for 30 min in EBSS starvation were immunoprecipitated with CHK2 antibody. The expression of p-CHK2 Thr68, CHK2, and ULK1 was detected by immunoblotting. (**G**) Lysates from HCT116 cells pretreated with CHK2 inhibitor (SIGMA, C3742, 20 μM) for 4 h and then treated with EBSS for 30 min were immunoprecipitated with CHK2 antibody. The expression of p-CHK2 Thr68, CHK2, and ULK1 was detected by immunoblotting. (**H**) Lysates from HCT116 cells expressing theindicated plasmids, treated or untreated with EBSS, were immunoprecipitated with FLAG antibody. The immunoprecipitates and lysates were analyzed by Western blot with the indicated antibodies. Quantitative analysis for the binding intensity of ULK1 are shown.

Our previous study found that metabolic stress can promote autophagy to maintain cellular homeostasis by activating CHK2. To further explore the molecular mechanism of CHK2 regulating autophagy, we screened CHK2 binding proteins through the candidate approach. We performed a co-immunoprecipitation (Co-IP) assay and showed that endogenous CHK2 co-precipitates with endogenous ULK1 (Figure 1A,B). Next, using an in vitro glutathione S-transferase (GST) pull-down assay, we found that CHK2 can directly bind to ULK1 (Figure 1C). Furthermore, enhanced binding between ULK1 and CHK2 was observed in response to metabolic and oxidative stress (Figure 1D,E). N-acetylcysteine (NAC) is a widely used oxygen radical scavenger. The interaction was significantly reduced by using NAC (Figure 1F), indicating that ROS are signaling molecules that facilitate the binding of CHK2 and ULK1. We found that the enhanced binding between these two proteins was accompanied by the activation of CHK2 Thr68. Next, CHK2 inhibitors significantly reduced its phosphorylation at Thr68 and the interaction between CHK2 and ULK1 under metabolic stress (Figure 1G). In addition, under metabolic stress, the binding between wild-type (WT) CHK2 and ULK1 was enhanced, whereas the binding of T68A CHK2 mutant to ULK1 was not. Mimic threonine 68 phosphorylation of CHK2 (T68D) promotes its interaction with ULK1, even under non-metabolic stress (Figure 1H). Taken together, the binding of CHK2 to ULK1 is dependent on the phosphorylation of CHK2 Thr68.

### 3.2. CHK2 Phosphorylates ULK1 at Ser556

**Figure 2:**
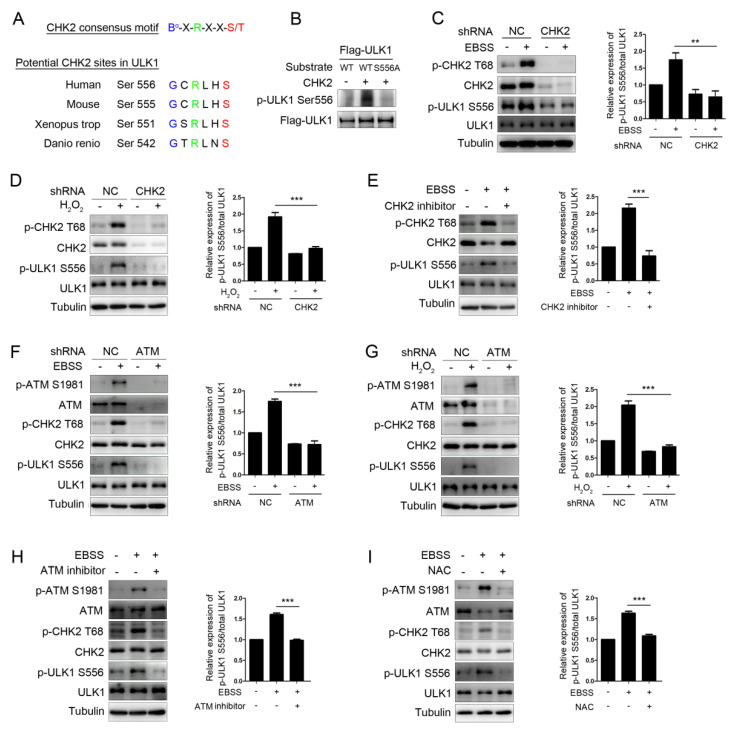
CHK2 phosphorylates ULK1 at Ser556. (**A**) Clustal alignment of the conserved sites in ULK1 matching the optimal CHK2 substrate motif. (**B**) In vitro CHK2 kinase assay using CHK2 recombinant human protein with FLAG-ULK1WT and FLAG-ULK1S556A as substrates, followed by immunoblotting analysis. (**C**,**D**) Western blot analysis with the indicated antibodies in H1299 cells with CHK2 knockdown. Cells were treated with EBSS (**C**) or H_2_O_2_ (500 μM) (**D**). (**E**) Western blot analysis with indicated antibodies in H1299 cells pretreated with CHK2 inhibitor for 3 h and treated with EBSS for 1 h. (**F**,**G**) Western blot analysis with indicated antibodies in H1299 cells with ATM knockdown. Cells were treated with EBSS (**F**) or H_2_O_2_ (500 μM) (**G**). (**H**) Western blot analysis with the indicated antibodies in H1299 cells pretreated with ATM inhibitor for 3 h and treated with EBSS for 1 h. (**I**) Western blot analysis with the indicated antibodies in H1299 cells pretreated with NAC for 4 h and treated with EBSS for 1 h. The results from three independent experiments are presented as mean ± SEM. ** *p* < 0.05, *** *p* < 0.001.

The protein modification of ULK1, especially the phosphorylation modification, is significant in its involvement in autophagy initiation. To determine whether CHK2 couldphosphorylate ULK1, we first utilized an optimal CHK2 substrate motif to search for possible amino acids sequences containing conservative candidate target sites in the ULK1 sequence [25]. ULK1 contains a Ser556 site matching the optimal CHK2 substrate motif conserved in higher eukaryotes (Figure 2A). Next, we further performed site-directed mutagenesis combined with an in vitro kinase assay and showed that CHK2 can phosphorylate ULK1 ser556, and that site-directed mutation of Ser556 to alanine (S556A) blocked the CHK2 mediated-ULK1phosphorylation on Ser556 (Figure 2B). Further, we observed that the phosphorylation of endogenous ULK1 Ser556 was enhanced under metabolic (Figure 2C) and oxidative stress (Figure 2D). However, in the CHK2 shRNA-treated cells, the phosphorylation of ULK1 Ser556 was significantly reduced. Likewise, small-molecule inhibitors of CHK2 can block the phosphorylation of ULK1 under metabolic stress (Figure 2E). Further, the phosphorylation of CHK2 and ULK1 was also reduced in ATM shRNA-treated or pharmacological ATM inhibition cells under both metabolic (Figure 2F) and oxidative stresses (Figure 2G). Taken together, these results demonstrate that the phosphorylation of ULK1 is dependent on the ATM/CHK2 signaling pathway in response to metabolic stress. Furthermore, the antioxidant NAC was able to block the activation of the ATM/CHK2/ULK1 signaling pathway under conditions of metabolic stress (Figure 2H). These results establish a critical redox-dependent role for the ATM-CHK2 signaling pathway in ULK1 Ser 556 phosphorylation under metabolic or oxidative stress.

### 3.3. CHK2-Mediated ULK1 Phosphorylation Promotes Autophagy

**Figure 3:**
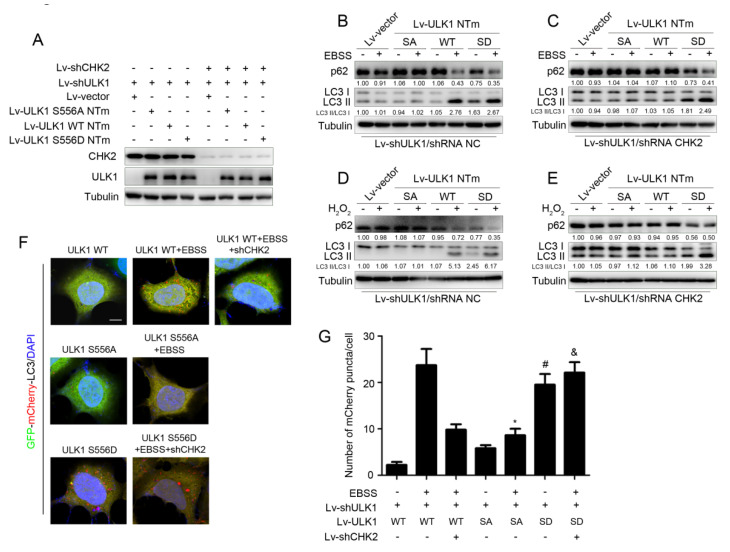
CHK2-mediated ULK1 phosphorylation promotes autophagy. (**A**) Western blot analysis with reconstituted expression of the indicated proteins in H1299 cells. (**B**,**C**) Western blot analysis of p62and LC3 in H1299 cells with reconstituted expression of ULK1 WT, S556A mutant, or S556D mutant treated with EBSS for 3 h with (**B**) or without (**C**) CHK2. (**D**,**E**) Western blot analysis of p62 and LC3 in H1299 cells with reconstituted expression of ULK1 WT, S556A mutant, or S556D mutant treated with H_2_O_2_ (500 μM) for 3 h with (**D**) or without (**E**) CHK2. (**F**) The red puncta is shown by representative confocal microscopic images in 293 cells expressing the indicated plasmids treated with EBSS for 2 h. (**G**) Data are presented as mean ± SEM. * ULK1 SA mutant treated with EBSS compared to ULK1 WT treated with EBSS, *p* < 0.001; # ULK1 SD mutant compared to ULK1 WT, *p* < 0.001 and & ULK1 SD mutant treated with EBSS without CHK2 compared to ULK1 WT treated with EBSS without CHK2, *p* < 0.001. Scale bar, 10 μm.

To further explore whether CHK2-mediated phosphorylation of ULK1 was involved in the regulation of autophagy, we constructed ULK1-depleted H1299 cell lines that stably expressed different mutants of ULK1 (WT, S556A, or S556D) with or without CHK2 (Figure 3A). Increased levels of autophagy were demonstrated by a decrease in the autophagy of substrate p62, and an increase in the conversion of LC3 from the non-lipidated form (LC3-I) to the phosphatidylethanolamine-bound form (LC3-II). ULK1 WT promoted metabolic and oxidative stress-induced autophagy, whereas increased autophagy was inhibited in cells lacking CHK2 expression. Compared with the effect of ULK1 WT, the ULK1 S556A mutant was not able to promote autophagy induced by metabolic and oxidative stress. However, the ULK1 S556D mutant promoted autophagy, even in cells that did not express CHK2 in the H1299 and HEK293 cell lines (Figure 3B–E and Supplementary Figure S1A–D).

Furthermore, we found similar conclusions by the quantitative analysis of autophagic flux, using tandemly labeled GFP-mCherry-LC3B. Given that mCherry fluorescence can be detected in both neutral autophagosomes and acidic autolysosomes, whereas GFP fluorescence is quenched in acidic autolysosomes, we can judge the extent to which autophagic flux proceeded and represented the promotion of autophagy levels, based on the reduction in yellow puncta and the appearance of only red puncta due to GFP quenching by fusion of autophagosomes with lysosomes. The red puncta representing autophagic flux were increased after the expression of ULK1 WT under metabolic stress, but not in CHK2 knockdown cells. Compared to the effect of ULK1 WT, the ULK1 S556A mutant failed to promote metabolic stress-induced autophagic flux. However, the ULK1 S556D mutant promotes autophagic flux, even in cells that did not express CHK2 (Figure 3F,G).

### 3.4. CHK2-ULK1-Mediated Autophagy Protects Cells against Metabolic Stress-Induced Cell Death

**Figure 4:**
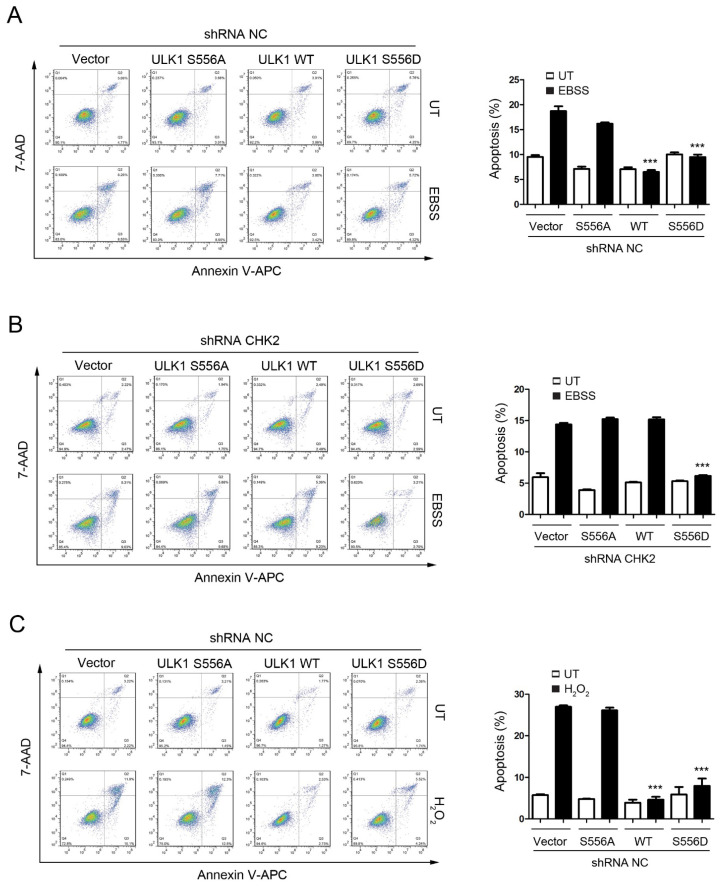

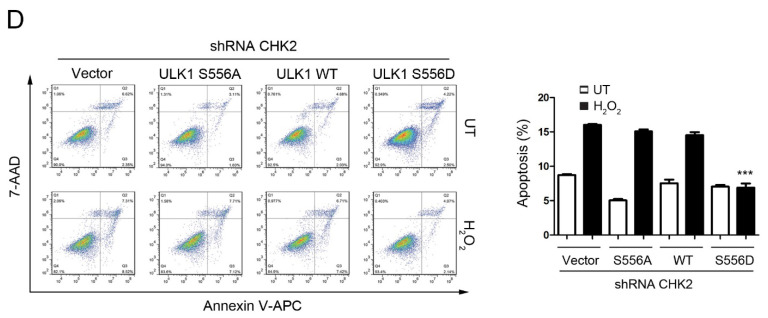
CHK2-ULK1 mediated autophagy protects cells against metabolic stress-induced cell death. (**A**,**B**) Flow cytometry analysis of apoptosis was performed in H1299 cells expressing the indicated plasmids treated with EBSS for 8 h with (**A**) or without (**B**) CHK2. The results from three independent experiments are presented as mean ± SEM. *** *p* < 0.001 compared to ULK1 S556A treated with EBSS. (**C**,**D**) Flow cytometry analysis of apoptosis was performed in H1299 cells expressing the indicated plasmids treated with H_2_O_2_ (500 μM) for 8 h with (**C**) or without (**D**) CHK2. The results from three independent experiments are presented as mean ± SEM. *** *p* < 0.001 compared to ULK1 S556A treated with H_2_O_2_ (500 μM) stimulation.

To test the role of CHK2-mediated phosphorylation of ULK1 in cell fate determination under stress conditions, we examined the effects of ULK1 WT, S556A, or S556D mutants on apoptosis, with or without CHK2 in the H1299 and HEK293 cell lines, in response to metabolic and oxidative stresses. The WT and S556D mutant reduced the number of cells undergoing apoptosis compared to the control, while the S556A mutant had no effect in response to metabolic and oxidative stress (Figure 4A,C, Supplementary Figure S2A,C). In addition, compared with CHK2-expressing cells, we found that apoptosis under stress was increased in CHK2-knockdown cells, even after expressing ULK1 WT (Figure 4B,D, Supplementary Figure S2B,D).

## 4. Discussion

More and more evidence indicates that ROS are considered to be signaling molecules that trigger autophagy under various stress conditions, but their specific regulatory mechanism is still largely unknown [6]. Here, we found that the autophagy-related protein ULK1 is a novel interacting protein of CHK2, and that binding is dependent on the accumulation of ROS caused by metabolic stress. CHK2 phosphorylates serine 556 of ULK1 to promote autophagy and inhibit cell apoptosis under metabolic stress. Our findings establish that the ATM/CHK2/ULK1 signaling pathway initiates an autophagic adaptive response by sensing ROS, and it protects cells from metabolic stress-induced cellular damage.

The relationship between ROS, autophagy, and apoptosis has been well revealed [4]. However, the possible regulatory mechanism is largely unknown. Our studies have revealed that ROS act as signaling molecules and activate the ATM/CHK2/ULK1 signaling pathway under metabolic and oxidative stress, while the ROS neutralizer NAC can inhibit the activation of the ATM/CHK2/ULK1 signaling pathway under metabolic stress. We further verified that CHK2-mediated ULK1 phosphorylation could promote autophagy and inhibit apoptosis in response to metabolic and oxidative stress. Previous studies have fully demonstrated the relationship between ROS, autophagy, and apoptosis. For example, the ROS neutralizer NAC can inhibit the accumulation of excessive ROS under metabolic stress, thereby inhibiting apoptosis. Autophagy can inhibit the accumulation of ROS by promoting the clearance of damaged mitochondria in response to metabolic stress, thereby inhibit apoptosis. Conversely, the inhibition of autophagy leads to the excessive accumulation of ROS under metabolic stress, and promotes apoptosis. As the molecular mechanisms of autophagy are well understood, the focus has shifted to the initiation signals of autophagy and the specific molecular mechanisms that initiate autophagy. More and more studies have demonstrated that various stimuli inducing autophagy could lead to an increase in ROS levels [26,27,28,29,30]. These findings suggest that ROS may play an essential role in autophagic initiation as a variety of central signals inducing autophagy. However, the molecular mechanism by which ROS initiates autophagy remains largely unknown. In recent years, DNA damage response (DDR)-transduced ataxia-telangiectasia mutant (ATM) has played a vital role in sensing ROS signaling and autophagy initiation. ATM activates TSC2 through the LKB1/AMPK signaling pathway to inhibit mTORC1, and this promotes autophagy [13]. Our previous study found that elevated levels of ROS induced by glucose deprivation and hypoxia can promote autophagy to maintain cellular homeostasis by activating the ATM/CHK2/Beclin 1 axis [10]. In this study, we further explored other molecular mechanisms by which the ATM-CHK2 signaling pathway initiated autophagy. We found that the autophagy-related protein ULK1 is an essential substrate of CHK2 under metabolic stress conditions, and further confirmed the role of the ATM/CHK2/ULK1 axis in autophagic initiation. This study further improved the function of the ATM-CHK2 signaling pathway to sense ROS signaling molecules in the autophagy regulatory network.

The formation of autophagosomes is a critical initial event in autophagy [31,32]. The serine/threonine-protein kinase ULK1 is the core protein involved in this step, and it forms a complex with FIP200, ATG13, and ATG101 to participate in the initiation of autophagy [33,34]. The phosphorylation of ULK1 is critical in autophagy regulation. Under adequate nutrient conditions, mTOR phosphorylates ULK1, resulting in the inactivation of the ULK1 complex. AMPK can promote autophagy by phosphorylating multiple serine/threonine sites in the intermediate domain of ULK1 (including serines 317, 467, 555, 637, 777 and threonine 574). We revealed that under oxidative stress conditions, CHK2 is activated by ATM, activated CHK2 binds to ULK1 and phosphorylates serine 556 of ULK1, and activated ULK1 promotes autophagy initiation and autophagic flux. For the first time, we revealed the specific molecular mechanism of how ROS signaling regulates ULK1 to promote autophagy, enriching the autophagy regulatory network of ULK1. ULK2 is highly homologous to ULK1, and is also particularly important in regulating autophagy. However, whether ULK2 is also involved in the autophagy process involving ROS signaling remains to be further investigated.

## 5. Conclusions

Our results show that the ROS–ATM–CHK2–ULK1–autophagy axis is a novel metabolic stress adaptive response pathway that protects cells from stress-induced cellular damage.

## Data Availability

Data is contained within the article and Supplementary Materials.

## Supplementary Materials

The following supporting information can be downloaded at: https://www.mdpi.com/article/10.3390/antiox11061166/s1, Figure S1: CHK2-mediated ULK1 phosphorylation promotes autophagy; Figure S2: CHK2-ULK1 mediated autophagy protects cells against metabolic stress-induced cell death.
