# Supplementary material for: CHK2 Promotes Metabolic Stress-Induced Autophagy through ULK1 Phosphorylation
Source: Antioxidants (Basel). 2022 Jun 14;11(6):1166. doi: 10.3390/antiox11061166 (PMC9219861; doi:10.3390/antiox11061166)
Supplement: Supplementary file 1 [file antioxidants-11-01166-s001.zip › antioxidants-1718541-supplementary.pdf]

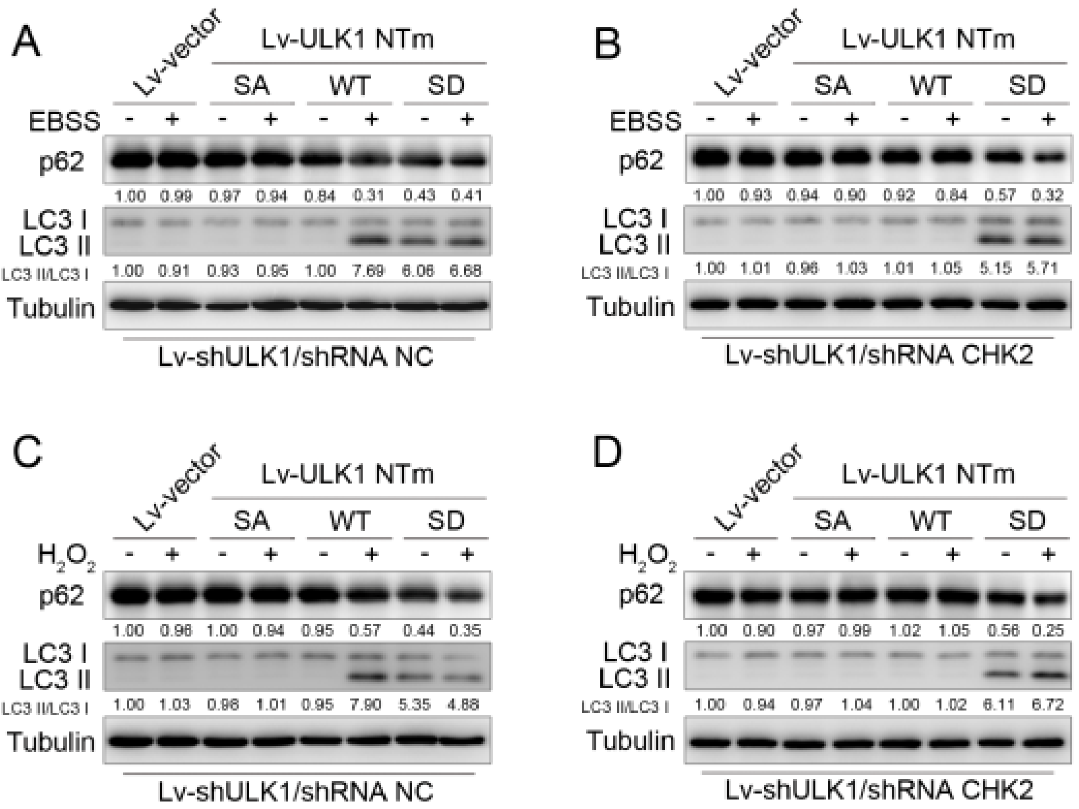

Figure S1. CHK2-mediated ULK1 phosphorylation promotes autophagy. (**A**,**B**) Western blot analysis of p62 and LC3 in HEK293 cells with reconstituted expression of ULK1 WT, S556A mutant, or S556D mutant treated with EBSS for 3 h with (**A**) or without (**B**) CHK2. (**C**,**D**) Western blot analysis of p62 and LC3 in HEK293 cells with reconstituted expression of ULK1 WT, S556A mutant, or S556D mutant treated with H_2O_2 (500 μM) for 3 h with (**D**) or without (**E**) CHK2.

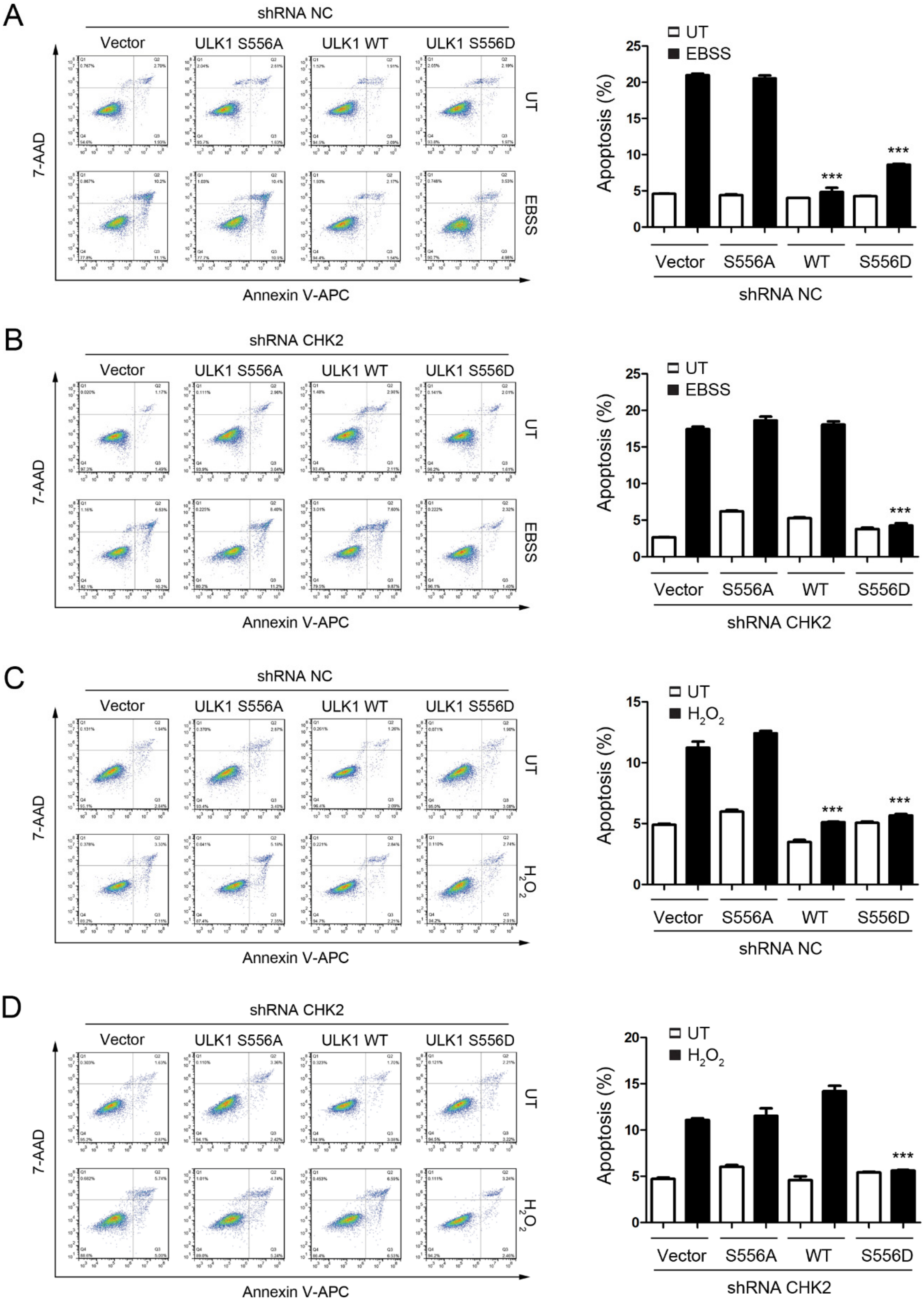

Figure S2. CHK2-ULK1 mediated autophagy protects cells against metabolic stress-induced cell death. (**A**,**B**) Flow cytometry analysis of apoptosis was performed in HEK293 cells expressing the

indicated plasmids treated with EBSS for 8 h with (**A**) or without (**B**) CHK2. The results from three independent experiments are presented as mean ± SEM. *** $p < 0.001$ compared to ULK1 S556A treated with EBSS. (**C**,**D**) Flow cytometry analysis of apoptosis was performed in HEK293 cells expressing the indicated plasmids treated with H_2O_2 (500 μM) for 6 h with (**C**) or without (**D**) CHK2. The results from three independent experiments are presented as mean ± SEM. *** $p < 0.001$ compared to ULK1 S556A treated with H_2O_2 (500 μM) stimulation.

Filename: antioxidants-1718541-supplementary.docx
Directory: E:\6.13\sensors-1740256
Template: C:\Users\MDPI\AppData\Roaming\Microsoft\Templates\Normal.dotm
Title:
Subject:
Author: guoqiqiang
Keywords:
Comments:
Creation Date: 5/17/2022 9:17:00 AM
Change Number: 224
Last Saved On: 6/14/2022 9:00:00 AM
Last Saved By: MDPI
Total Editing Time: 2,611 Minutes
Last Printed On: 6/14/2022 2:10:00 PM
As of Last Complete Printing
Number of Pages: 3
Number of Words: 195 (approx.)
Number of Characters: 982 (approx.)
